# Microbial dispersal into surface soil is limited on a meter scale

**DOI:** 10.1093/ismejo/wraf169

**Published:** 2025-08-05

**Authors:** Kendra E Walters, Kristin M Barbour, John M Powers, Jennifer B H Martiny

**Affiliations:** Department of Ecology and Evolutionary Biology, University of California, Irvine, Irvine, CA 92697, United States; Biology Department, Reed College, Portland, OR 97202, United States; Department of Ecology and Evolutionary Biology, University of California, Irvine, Irvine, CA 92697, United States; Department of Ecology and Evolutionary Biology, University of California, Irvine, Irvine, CA 92697, United States; Department of Ecology and Evolutionary Biology, University of California, Irvine, Irvine, CA 92697, United States

**Keywords:** dispersal limitation, dispersal rate, microbial communities, soil

## Abstract

Dispersal shapes microbial communities, yet it is largely unknown how fast or how far free-living microorganisms move in the environment. Here, we deployed microbial traps along transects spanning a grassland and neighboring shrubland to quantify the rate and distance at which microorganisms disperse into the soil surface. We found that bacteria disperse at a similar rate across the two ecosystems, and both bacteria and fungi exhibit a signature of dispersal limitation at a meter scale, indicating highly heterogeneous dispersal of microorganisms into soil.

Although dispersal has long been recognized as a key process in the assembly of plant and animal communities, its role in shaping microbial communities has only recently been established [1–3]. Directly tracking the movement of individual microorganisms in the field remains a technical challenge. As such, the fundamental properties of microbial dispersal, including the rate and distance that free-living microbes move in their environments, are largely unknown.

Dispersal rate, or the number of individuals immigrating into a defined area per unit time, is an important determinant of how strongly dispersal impacts community structure (species abundance and composition) relative to other processes, such as environmental selection [4, 5]. Using microbial “traps” (e.g. filter paper, glass slides, or air samplers), previous studies have quantified the rate at which bacteria and fungi disperse in aquatic [6], aerial [7], and terrestrial [8, 9] systems. However, these measurements remain rare for bacteria, and it is unclear the degree to which dispersal rates vary across space and thus may contribute to the biogeography of microbial communities.

To investigate the spatial variability of microbial dispersal, we quantified the rate of bacterial immigration along 30-m transects spanning two ecosystems, a grassland and shrubland (Supplementary methods, Fig. 1a). We placed two types of glass slides on the soil surface, sterile slides open to dispersal (accumulation rate samples, *n* = 96) and slides closed to dispersal (death rate samples, *n* = 35) and collected the slides over 2 months (Fig. 1b and c). Using flow cytometry, we measured the number of bacterial cells accumulating on the open glass slides and the number of cells declining on the death rate samples over time. The glass slides capture cells moving into the surface soil from different sources (e.g. soil, vegetation, or the atmosphere) and physical vectors (e.g. wind or rain), but provide no nutrients for growth. Therefore, the number of cells accumulating on the open slides (Fig. 2a) reflects the immigration rate minus the death rate of cells (see Supplementary methods). To quantify cell death, death rate samples were inoculated with a microbial community extracted from either grassland or shrubland leaf litter before being placed in the field. Although death rates may vary between different source communities, leaf litter was previously demonstrated to be a major source of microbes immigrating into the surface soil at this site [9]. Additionally, ecosystem-specific litter was used, because microbial composition varies between the grassland and shrubland [10].

**Figure 1:**
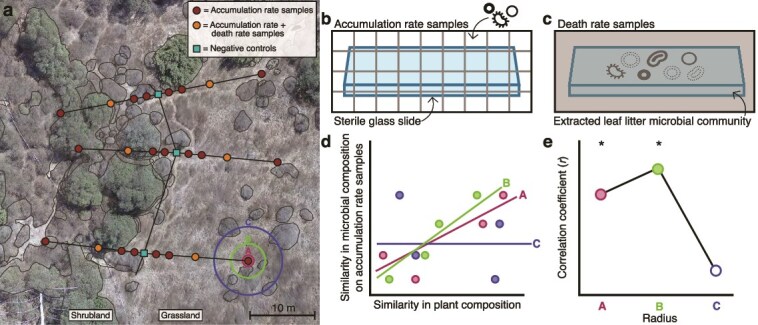
Experimental approach. (a) An aerial photo of the field site showing the three transects spanning the shrubland and grassland boundary (dashed line). Negative controls (sterile glass slides closed to dispersal) were placed at the ecosystem boundary along each transect (*n* = 18 total). Polygons drawn around shrubs, forbs, and bare soil were used in conjunction with average grass composition to calculate overall plant community composition at increasing radii from each sampling location (e.g., a, b, c). (b) Accumulation rate samples (sterile glass slides open to dispersal) were placed at eight locations along the transects: 1, 3, 7, and 15 m into each ecosystem (*n* = 48 per ecosystem). (c) Death rate samples (sterile slides with a known number of cells and closed to dispersal) were deployed at two locations per transect (7 m into each ecosystem, *n* = 17–18 per ecosystem). (d) Hypothetical relationship between similarity in plant composition and similarity in the composition of microbes dispersing onto accumulation rate samples. Each point represents a pairwise comparison between two accumulation rate samples. Symbol color represents the radius at which plant composition was measured from the samples. Lines depict the correlation at each sampling radius. (e) Hypothetical correlation coefficients (*r*) from panel D. Significant relationships are depicted by filled circles. The strength of the correlation coefficients reveals the scale at which plant composition most strongly contributes to the identity of immigrating taxa.

**Figure 2:**
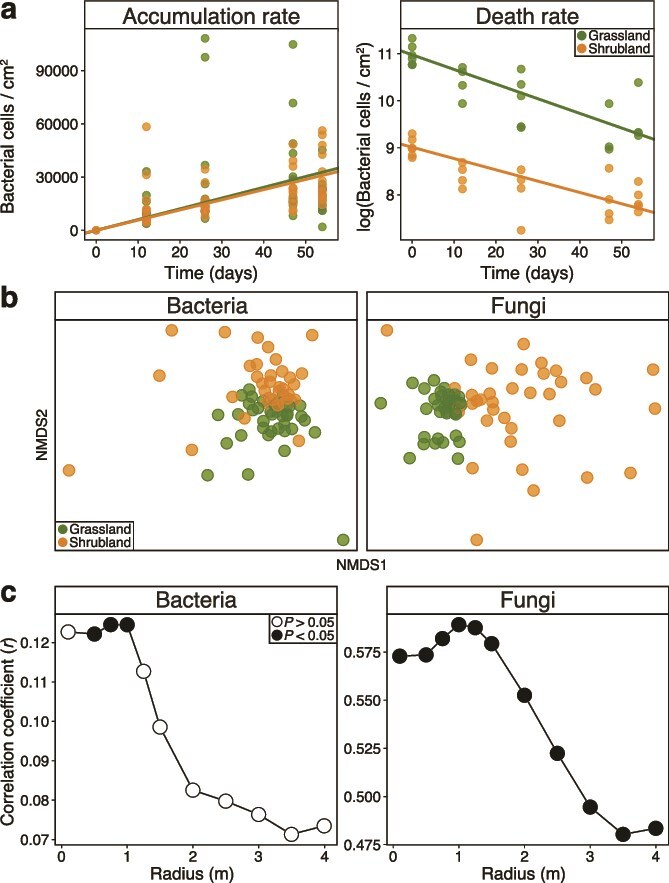
Microbial dispersal rates and distance. (a) Bacterial abundance on the accumulation and death rate slides (natural log) over time. Lines are linear regressions for accumulation and death rates, respectively. The average slope of the death rate linear regressions is used to model immigration rates. (b) Non-metric multidimensional scaling (NMDS) ordination of bacterial and fungal community composition captured on the accumulation rate slides. (c) Mantel correlation coefficients between bacterial and fungal composition and the surrounding plant composition, measured within circles of increasing radii around each sample. Each point represents one partial mantel test.

Death rate did not differ significantly between the grassland and shrubland (Table S1; ANCOVA: Time × Ecosystem interaction *P* value = .17) with 2.76% of the community dying on average per day across the landscape (Fig. 2a). Accumulation rate, or the number of intact cells (detected by flow cytometry) captured on the open slides over time, also did not differ between ecosystems (Fig. 2a; Table S2; *P* value = .61). Assuming a dynamic relationship between immigration and death rates (see Supplementary methods), the rate of bacterial dispersal into the soil surface averaged 1060 ± 90 cells/cm^2^/day (±SE). This rate represents 0.04% of the average bacterial abundance found in leaf litter at this research site (~2.8 million cells/cm^2^). The relatively small fraction of cells moving into the topsoil per day suggests that dispersal is likely not homogenizing the bacterial community on the soil surface (i.e. mass effects [4]).

Immigration rate captures how fast cells move into the surface soil, but it does not indicate how far cells are moving—i.e. the degree to which cells are dispersal limited. Dispersal limitation is critical for predicting the spread and gene flow of a specific microbial species, such as a pathogen [11], as well as the successional dynamics and biogeography of microbial communities as a whole [12]. In plants, dispersal limitation is commonly reported as a dispersal kernel: a probability density function describing the likelihood that seed deposition will occur at certain distances from a parent plant [13]. Dispersal kernels have been characterized for a few fungal species [8, 14, 15] and reveal that the degree of dispersal limitation of fungal spores ranges widely across species. These studies have focused on species that are host-associated, allowing for their abundance to be quantified at increasing distances from a known point source. In contrast, characterizing the dispersal kernel of free-living microorganisms adds another layer of complexity, because they may be dispersing simultaneously from many sources.

To address the challenge of tracking individual microbes, we used a community approach to estimate dispersal limitation in the field by measuring how the composition of dispersing microbes was impacted by surrounding vegetation, an important source of dispersal at our field site [16]. The composition of both bacteria and fungi on the accumulation rate slides were distinct between the grassland and shrubland (Fig. 2b; Table S3; PERMANOVA: *P* value ≤.001; estimated variance explained by ecosystem: 3% and 18% for bacteria and fungi respectively). Within ecosystems, fungal, but not bacterial, composition on the slides was more variable in the shrubland than that in the grassland (Table S3; PERMDISP: *P* value <.001), potentially reflecting greater heterogeneity of plant composition in the shrubland. These differences in the composition of microbes dispersing into the soil indicates that some bacterial and fungal cells are dispersal limited at this study’s scale, within the 30 m transects.

Given that the grassland and shrubland are immediately adjacent with no major differences in slope, aspect, soil type, or climate [17], we hypothesized that shifts in plant community composition between the ecosystems underlie these distinct dispersal communities. To test the spatial scale at which plant composition most strongly contributes to the identity of immigrating taxa, we measured the correlation between microbial composition on the accumulation rate slides and plant composition within 11 increasingly large circles (Fig. 1d and e), with radii ranging from 0.1 to 4 m away from the slides. When controlling for variation explained by geographic distance among samples, the strength of the correlation between the microbial composition on the open slides and surrounding plant composition was highest at around 1 m for both bacteria and fungi (Fig. 2c; Table S4; Mantel’s *r* = 0.13 and *r* = 0.59, respectively). This highly local signal of dispersal limitation corroborates the strong compositional differences in dispersing microbes between the grassland and shrubland (Fig. 2b). The correlation was also much stronger for fungi than bacteria at all radii. This result may reflect stronger relationships between plants and fungi or alternatively, shorter average dispersal distances of fungi compared to bacteria [18]. Indeed, a SourceTracker analysis of potential dispersal sources (air, plant litter, and soil) suggests that dispersal from leaf litter contributed more to the fungal communities captured on the accumulation slides compared to the bacterial community (Fig. S1).

Together, our results quantify fundamental properties of microbial dispersal in a terrestrial ecosystem. Bacteria disperse into the soil surface at a similar, albeit relatively low, rate across a landscape. This rate seems to be determined by abiotic conditions (e.g. wind speed, precipitation, and landscape topography) that are shared between the grassland and shrubland [19, 20]. Even though overall dispersal rates were not spatially variable, the composition of dispersing microbes was highly localized, with a signature of dispersal limitation detected at a meter scale for both bacteria and fungi. This result does not mean that individual microorganisms are not moving much longer distances, but that enough are restricted to shorter distances to generate these patterns. This local signature indicates that dispersal may interact with other eco-evolutionary processes, like genetic and ecological drift, to shape biodiversity and biogeography of microbial communities even at a meter scale [21].

## Supplementary Material

Supplementary_information_wraf169

## Data Availability

Sequence data generated from this work are available in the NCBI database under the BioProject Accession number PRJNA1216276. All data and code used to produce analyses and figures are available at https://github.com/kendraewalters/landscape_dispersal.

## References

[1] Vannette RL, Fukami T. Dispersal enhances beta diversity in nectar microbes. *Ecol Lett* 2017;20:901–10. 10.1111/ele.12787 28597955

[2] Zha Y, Berga M, Comte J. et al. Effects of dispersal and initial diversity on the composition and functional performance of bacterial communities. *PLoS One* 2016;11:e0155239. 10.1371/journal.pone.0155239 27182596 PMC4868275

[3] Peay KG, Garbelotto M, Bruns TD. Evidence of dispersal limitation in soil microorganisms: isolation reduces species richness on mycorrhizal tree islands. *Ecology* 2010;91:3631–40. 10.1890/09-2237.1 21302834

[4] Shmida A, Wilson MV. Biological determinants of species diversity. *J Biogeogr* 1985;12:1–20. 10.2307/2845026

[5] Mouquet N, Loreau M. Community patterns in source-sink metacommunities. *Am Nat* 2003;162:544–57. 10.1086/378857 14618534

[6] Jones SE, Newton RJ, McMahon KD. Potential for atmospheric deposition of bacteria to influence bacterioplankton communities. *FEMS Microbiol Ecol* 2008;64:388–94. 10.1111/j.1574-6941.2008.00476.x 18393990

[7] Egan C, Li DW, Klironomos J. Detection of arbuscular mycorrhizal fungal spores in the air across different biomes and ecoregions. *Fungal Ecol* 2014;12:26–31. 10.1016/j.funeco.2014.06.004

[8] Peay KG, Schubert MG, Nguyen NH. et al. Measuring ectomycorrhizal fungal dispersal: macroecological patterns driven by microscopic propagules. *Mol Ecol* 2012;21:4122–36. 10.1111/j.1365-294X.2012.05666.x 22703050

[9] Walters KE, Capocchi JK, Albright MBN. et al. Routes and rates of bacterial dispersal impact surface soil microbiome composition and functioning. *ISME J* 2022;16:2295–304. 10.1038/s41396-022-01269-w 35778440 PMC9477824

[10] Finks SS, Weihe C, Kimball S. et al. Microbial community response to a decade of simulated global changes depends on the plant community. *Elem Sci Anthr* 2021;9:00124. 10.1525/elementa.2021.00124

[11] Brown JKM, Hovmøller MS. Aerial dispersal of pathogens on the global and continental scales and its impact on plant disease. *Science* 2002;297:537–41. 10.1126/science.1072678 12142520

[12] Fierer N, Nemergut D, Knight R. et al. Changes through time: integrating microorganisms into the study of succession. *Res Microbiol* 2010;161:635–42. 10.1016/j.resmic.2010.06.002 20599610

[13] Nathan R, Muller-Landau HC. Spatial patterns of seed dispersal, their determinants and consequences for recruitment. *Trends Ecol Evol* 2000;15:278–85. 10.1016/S0169-5347(00)01874-7 10856948

[14] Golan JJ, Pringle A. Long-distance dispersal of fungi. *Microbiol Spectr* 2017;5. 10.1128/microbiolspec.funk-0047-2016 PMC11687522 28710849

[15] Rieux A, Soubeyrand S, Bonnot F. et al. Long-distance wind-dispersal of spores in a fungal plant pathogen: estimation of anisotropic dispersal kernels from an extensive field experiment. *PLoS One* 2014;9:e103225. 10.1371/journal.pone.0103225 25116080 PMC4130500

[16] Barbour KM, Barrón-Sandoval A, Walters KE. et al. Towards quantifying microbial dispersal in the environment. *Environ Microbiol* 2022;25:137–42. 10.1111/1462-2920.16270 36308707 PMC10100412

[17] Potts DL, Suding KN, Winston GC. et al. Ecological effects of experimental drought and prescribed fire in a southern California coastal grassland. *J Arid Environ* 2012;81:59–66. 10.1016/j.jaridenv.2012.01.007

[18] Zhang G, Wei G, Wei F. et al. Dispersal limitation plays stronger role in the community assembly of fungi relative to bacteria in rhizosphere across the arable area of medicinal plant. *Front Microbiol* 2021;12:713523. 10.3389/fmicb.2021.713523 34484152 PMC8415459

[19] Maltz MR, Carey CJ, Freund HL. et al. Landscape topography and regional drought alters dust microbiomes in the Sierra Nevada of California. *Front Microbiol* 2022;13:856454. 10.3389/fmicb.2022.856454 35836417 PMC9274194

[20] Tignat-Perrier R, Dommergue A, Thollot A. et al. Global airborne microbial communities controlled by surrounding landscapes and wind conditions. *Sci Rep* 2019;9:14441. 10.1038/s41598-019-51073-4 31595018 PMC6783533

[21] Martiny JBH, Martiny AC, Brodie E. et al. Investigating the eco-evolutionary response of microbiomes to environmental change. *Ecol Lett* 2023;26:S81–90. 10.1111/ele.14209 36965002

