# Supplementary material for: Microbial dispersal into surface soil is limited on a meter scale
Source: ISME J. 2025 Aug 5;19(1):wraf169. doi: 10.1093/ismejo/wraf169 (PMC12393145; doi:10.1093/ismejo/wraf169)
Supplement: Supplementary_information_wraf169 [file supplementary_information_wraf169.pdf]

Supplementary Information

Microbial dispersal into surface soil is limited on a meter scale.

Kendra E. Walters[1,2], Kristin M. Barbour[1#], John Powers[1], Jennifer B.H. Martiny[1]

[1]Department of Ecology and Evolutionary Biology, University of California Irvine, Irvine, CA USA

[2]Biology Department, Reed College, Portland, OR, USA

# Address correspondence to Kristin M. Barbour (kbarbou1@uci.edu)

Supplementary Information

Supplementary Methods

Field site

The experiment was conducted adjacent to the Loma Ridge Global Change Experiment in California, USA (33°44′ N, 117°42′ W, 365 m elevation) from June 18th, 2020 – August 11th, 2020. The site encompasses a Mediterranean grassland and shrubland, which experiences dry warm summers and cool wet winters, with 325 mm mean annual precipitation and 17°C mean temperature. The grassland is dominated by non-native grasses *Bromus* sp. and *Avena* sp., non-native forbs *Hirschfeldia incana* and *Erodium* sp., and the native forb *Deinandra fasciculata*. The shrubland is dominated by native shrubs *Salvia mellifera*, *Malosma laurina,* and *Artemisia californica*, native herb *Acmispon glaber*, and invasive grass *Bromus madritensis* [1, 2].

Experimental Approach

To identify the taxa dispersing across the landscape, we deployed dispersal "traps" at eight locations along three 30 m transects that spanned the boundary between the grassland and shrubland ecosystems (Fig. 1A). Accumulation rate samples (n = 96) were placed on top of the soil 1, 3, 7, and 15 m into each ecosystem and, thus, captured microorganisms immigrating onto the soil surface (see *Accumulation rate slides* and *Sample collection*). To estimate bacterial dispersal rate, we measured the number of bacterial cells that accumulated on the open glass slides over time; however, the cell abundance on these samples is affected by both immigration and death rates. Therefore, to estimate immigration rate, we simultaneously deployed samples that allowed us to calculate in-field death rates. These death rate samples (n = 47) contained a known number of bacterial cells and were closed to immigration, allowing us to measure the decrease in abundance over time (see *Death rate slides*). Death rate samples were deployed at

two locations along each of the three transects (7 m into each ecosystem) so that we could measure death rate independently in the grassland and shrubland. In addition, we deployed in-field negative control samples (n = 18) at the midpoint of each transect which consisted of a sterile glass enclosed in nylon with 0.22 µm pores that blocked immigration. These negative controls provided a measure of potential contamination into the death rate samples. All samples were set out in the field on June 18, 2020 and were secured under chicken wire and staked into the ground to prevent disturbance (see *Accumulation rate slides* and *Death rate slides*).

Plant composition and geographic sampling locations

We characterized the plant composition and geographic sampling locations by taking aerial drone (Holy Stone, HS100) photos of the study area. Photos were stitched together using Hugin (2019.2.0) and aligned to aerial images of the field site to ensure accurate aspect ratio. Sampling locations were identified on the composite aerial photo by the presence of red flagging, and polygons were drawn around individual shrubs or forbs and areas of exposed soil. Plant polygons around each sampling location along the three transects were identified to species in the field, including the species composition of the grassland which was applied to all areas of grass coverage. Plant composition was calculated at increasing radii around each sampling location (see *Sample collection)* as the proportion of area covered by shrub or forb species (plant polygons), grass, or exposed soil (Fig. 1A, see *Statistical analyses*).

Accumulation rate slides

To characterize the abundance and composition of cells immigrating into the soil surface, 96 sterile glass microscope slides were sealed within bags (5 cm x 10 cm) constructed from

window screen with 2 mm pores. The 2 mm pores in the window screen allow bacterial and fungal cells to immigrate onto the accumulation rate slides in the field. Prior to being deployed into the field, glass microscope slides (2.5 cm x 7.5 cm) were sterilized in 70% ethanol, dried, sealed into bags, and autoclaved. Four accumulation rate slides were placed at each of the eight locations along the three transects (3 transects x 8 locations x 4 replicates = 96 samples).

Death rate slides

To measure bacterial death rate, 47 glass slides containing a known number of bacterial cells were created using leaf litter collected from the grassland and shrubland at the field site. We used bacterial communities derived from leaf litter on the soil surface to keep the taxonomic composition consistent with the rest of the experiment. To create the death rate slides, bacterial cells were extracted separately from both grass and shrub litter by steeping the litter in 1 L of 0.9% saline solution overnight. The leaf litter was then filtered through cheesecloth, and the filtrate was aliquoted in 50 mL volumes, then further concentrated and washed by pelleting the cells and resuspending into 0.5 mL sterile 0.9% saline solution. Aliquots of 100 µL of either the grass- or shrub-derived cells were then spread onto ethanol-sterilized glass microscope slides and allowed to dry before being sealed into autoclaved nylon bags (0.22 µm pores) that were closed to dispersal (Fig. 1c). The slides were stored at room temperature overnight until being deployed in the field. Death rate slides made with grass-derived cells were deployed in the grassland (n = 17) and those made with shrub-derived cells were deployed in the shrubland (n = 18). The 12 samples that were not deployed into the field (6 replicates per ecosystem) were used to measure initial bacterial abundance on the death rate slides (see *Bacterial abundance*).

Supplementary Information

Sample collection

At four timepoints, we collected 4 accumulation rate slides and 1-2 death rate slides per ecosystem from each of the three transects (totaling 12 accumulation rate slides and 4-5 death rate slides per ecosystem per timepoint). At each timepoint, 1-2 negative controls were also collected from the midpoint of each transect (totaling 4-5 negative control slides per timepoint). Samples were collected after approximately 2 weeks (June 30th, 2020), 4 weeks (July 14th, 2020), 7 weeks (August 4th, 2020), and 8 weeks (August 11th, 2020) in the field. On the day of collection, glass slides were transferred into sterile Wirl-Paks (Nasco, WI, USA) containing 2 mL of 0.9% sterile saline. The Whirl-paks were agitated by hand for 30 s to dislodge microbial cells from the surface of the glass slide into the saline solution. 500 µL of this cell solution was removed and stored at -70ºC until DNA extractions. After this aliquot was removed, 167 µL of 10% phosphate-buffered GTA was added to bring the solution to a final concentration of 1% phosphate-buffered GTA. Samples were filtered through a 4 µm pore size vacuum filter to remove large particulates and stored in the dark at 4°C overnight for bacterial abundance measurements.

Dispersal source sampling

To characterize potential sources of microbial dispersal into the surface soil [3], we collected surrounding soil, leaf litter, and air samples from the field site at the second collection timepoint (July 14th, 2020). Soil samples were obtained using a sterile scoop to collect approximately 2 g of surface soil at four locations along each of the three transects (n = 12; 6 per ecosystem). To collect plant litter, approximately 3 g of leaf litter was collected by hand or with sterile clippers at the same locations along the transects where surface soil was sampled (n = 12;

6 per ecosystem). To collect air samples and sterile glass slides (n = 3) open to dispersal (sealed within window screen bags with 2 mm pores) and Whatman Mini and FTA cards (n = 3) were elevated 3 m above the soil surface on a weather tower located at the field site. Air samples were deployed on June 18th and then collected on July 14th. Aliquots of 0.05 g leaf litter, 0.1 g soil, 250 µL glass slide, and half of the FTA card were taken on the day of sample collection and stored at -70ºC until DNA extraction.

Bacterial abundance

Bacterial abundance from the accumulation rate, death rate, and negative control slides was measured using flow cytometry [4]. To process samples on a NovoCyte flow cytometer (ACEA Biosciences, San Diego, CA, USA), 2 µL of SYBR Green (200x, ThermoFisher, Waltham, Massachusetts, USA) was added to 400 µL of the GTA-fixed filtrate and then incubated in the dark at room temperature for 10 minutes. Samples were run for 30 seconds at 40 µL/min, using a SYBR-Green-H threshold value of 1,500 and side scatter height threshold value of 1,000. Gating parameters used to count particles in the size of typical bacterial cells were previously optimized [4]. Cell abundance for all samples was calculated as the number of stained counts minus counts from a negative control buffer. For the death rate samples, we controlled for any potential contamination by also subtracting the average number of cells captured on the negative glass slide controls in the field. Negative controls accumulated few cells in the field (198 ± 78 cells/cm^2 on average ± SE) compared to the accumulation rate slides (22,714 ± 2,048 cells/cm^2 on average ± SE), demonstrating that dispersal was significantly reduced into the death rate slides. Given that the negative controls had such low abundance, DNA was not extracted from these samples.

Supplementary Information

Amplicon sequencing

Genomic DNA was extracted from 250 µL of the glass slide saline solution and half of each FTA card (air sample) using ZymoBIOMICS Microprep DNA Extraction Kit with tubes. DNA was also extracted from the 0.05 g leaf litter and 0.1 g soil aliquots using the ZymoBIOMICS Microprep DNA Extraction Kit with plates. The kit protocols were followed with the following modifications. For the glass slide and air samples, bead beating was reduced to 3 minutes at 6.5 m/s in a FastPrep 24 (MP Biomedicals, Irvine CA, USA), instead of 5 minutes, to avoid shearing the DNA in these low biomass samples. For all tube extraction samples, the Zymo-Spin III-F filter was not used, and water was heated to 60°C for elution. For the leaf litter and soil plate extractions, maximum centrifuge speed was 2808 x g, instead of 3500 x g, and centrifuge time was increased from 3 min to 4 min and 5 min to 7 min when applicable.

To characterize the bacterial community, we amplified the V4 – V5 region of the 16S ribosomal RNA gene using the 515F (GTGYCAGCMGCCGCGGTAA) – 926R (CCGTCAATTCCTTTRAGTTT) primers [5, 6]. For 16S rRNA gene PCR reactions, 5 µL of genomic DNA from the glass slide and air samples was combined with 12.5 µL of AccustartII PCR ToughMix (Quanta BioSciences Inc, Beverly, MA, USA), 0.5 µL of the 10 µM barcoded forward primer, 0.5 µL of the 10 µM reverse primer, and 6.5 µL PCR grade water. For the high biomass leaf litter and soil samples, 1 µL of genomic DNA was added with 10.5 µL PCR grade water. Following an initial denaturation step at 94°C for 3 min, the PCR was cycled 30 times at 94°C for 45 s, 55°C for 30 s, and 72°C for 60 s, with a final extension at 72°C for 10 min. Because 3 glass slides did not amplify with the above protocol, the PCR was repeated for those samples with 8 µL of template DNA (final volume of 25 µL) and 34 cycles. No 16S rRNA gene

amplicons were produced from the air samples even when repeating PCR with 8 μL of template DNA and were, therefore, removed from all downstream analyses.

To characterize the fungal community, we amplified the ITS2 region of the Internal Transcribed Spacer (ITS) using the ITS9F (GAACGCAGCRAAIIGYGA) – ITS4 (TCCTCCGCTTATTGATATGC) primer combination [7]. For ITS PCR reactions, 5 μL of genomic DNA from the glass slide and air samples was combined with 12.5 μL of AccustartII PCR ToughMix (Quanta BioSciences Inc, Beverly, MA, USA), 0.75 μL of the 10 μM barcoded forward primer, 0.75 μL of the 10 μM reverse primer, and 6 μL PCR grade water. For the leaf litter and soil samples, 1 μL of genomic DNA was added with 10 μL PCR grade water. After an initial denaturing step at 94°C for 3 min, the PCR was cycled 35 times at 95°C for 45 s, 50°C for 60 s, and 72°C for 90 s, with a final extension at 72°C for 10 min.

To prepare libraries for sequencing, PCR products were pooled at different volumes based on band brightness on gel pictures (high (1 μL), medium high (2 μL), medium (3 μL), medium weak (4 μL), weak (5 μL), and none (10 μL)). All samples were pooled together for one sequencing run – both 16S rRNA gene and ITS amplicons. After pooling, the library was purified using SpeedBeads magnetic carboxylate-modified particles (GE Healthcare UK Limited) and sequenced in a paired-end MiSeq System (Illumina) run (2 x 250 bp) at the Genomics High Throughput Facility, UC Irvine, Irvine, CA, USA.

Bioinformatic processing and analysis

Sequence data were processed in QIIME2 version 2023.7 [8]. The forward reads were trimmed to 5 – 224 base pairs of the 16S rRNA gene segment and 5 – 235 base pairs of the ITS segment. The reverse reads were discarded due to low quality. We used DADA2 [9] to define

operational taxonomic units (OTUs) defined at 100% identity (exact sequence variants). Taxonomy was assigned using the q2-feature-classifier plugin and classify-sklearn in QIIME 2 [10] to generate a Naïve Bayes classifier trained on reference sequences from the SILVA 138 SSU Ref NR99 database at a 99% OTU identity trimmed to 224 bp for bacteria [11] and untrimmed UNITE database (v.10.0) for fungi [12].

Statistical analyses

We accounted for differences in sequencing depth by rarefying all samples to 970 sequences (bacterial communities) and 1,840 sequences (fungal communities) with 1,000 resamplings. These lower rarefaction levels are expected for low abundance glass slide samples [3, 13]. For each resampling, we calculated a Bray-Curtis dissimilarity matrix, taking the median similarity values of all 1,000 matrices using the 'vegan' package in R [14, 15]. To test for differences in community composition on the accumulation rate slides between ecosystem, timepoint, and their interaction, we used this median matrix to perform a permutational multivariate analysis of variance (PERMANOVA) and post hoc tests using PERMANOVA+ on PRIMER version 6 [16]. The PERMANOVA model was run as type III partial sum of squares for 999 permutations. Variance in community composition explained by experimental factors was calculated by dividing the estimated components of variance of statistically significant terms by the sum of all significant terms and residuals. Dispersal is also known to influence β-diversity, or variation in community composition. Therefore, we performed a test of the homogeneity of dispersion (PERMDISP) to assess whether dispersal differentially altered β-diversity between ecosystems and across timepoints. The proportion of the bacterial and fungal

communities captured on the accumulation rate samples attributed to distinct dispersal sources (air, surrounding leaf litter, and surface soil) was estimated using SourceTracker2 [17].

Given that bacterial accumulation (abundance on the glass slide) is a function of immigration and death rates, we first calculated in-field death rates as the slope of a linear model fitted to the decline in bacterial abundance (natural log) on the death rate slides over time. Linear models were performed using the "lm" function from the stats package in R. Death rates were initially calculated separately in the grassland and shrubland ecosystems. To test whether death rate differed by ecosystem, we used an analysis of covariance (ANCOVA) to test the influence of ecosystem, time (represented by days in the field since the experiment began), and a time-by-ecosystem interaction on bacterial abundance on the death rate slides. Here, a significant time-by-ecosystem interaction would indicate different death rates between ecosystems. Death rates did not significantly differ between ecosystems (ANCOVA: P value = 0.17). We, thus, used the average in-field death rate between the grassland and shrubland for all downstream analyses. After calculating in-field death rate, we then calculated immigration rates assuming a dynamic relationship between accumulation and death rates (not at equilibrium). This equation modeled bacterial abundance on the glass slides (n) as $n(t) = \frac{i}{d} \times (1 - e^{-d \times t})$, where d is the average in-field death rate, t is time (days in the field), and i is the immigration rate estimated using the "nls" function in the stats package in R. This equation is derived by integrating $\frac{dn}{dt} = i - d * n(t)$ when forced through the origin. Because the number of cells accumulating on the open glass slides (accumulation rate) and number of cells declining on the death rate samples (death rates) did not vary significantly between ecosystems, we calculated a single immigration rate (i) for the entire landscape.

Supplementary Information

To compare the relative size of the immigrating community to the resident community on the soil surface, we compared the average number of bacterial cells immigrating into the soil surface (per day per cm^2) to the average number of bacterial cells on leaf litter (per cm^2). Bacterial abundance was previously measured from a thin layer of grass and shrub litter collected from the grassland and shrubland at Loma Ridge (unpublished data). Leaf litter was collected, deployed back into the field in nylon litter bags, and collected after 5-, 10-, and 18-months in the field to capture different stages of decomposition. Bacterial abundance was assessed using flow cytometry as previously described [4, 13] and then averaged between ecosystems and timepoints to provide a general estimate of bacterial abundance in the leaf litter layer at this field site.

To examine the scale at which local vegetation most strongly contributes to microbial dispersal, we tested how geographic distance and plant composition influenced the composition of bacteria and fungi immigrating onto the open slides using a Mantel correlogram [18, 19]. Geographic distance was measured as the pairwise distance among locations. Plant composition was initially measured by calculating the proportion of area covered by each plant species (outlined polygons) within a 1 m radius around each sampling location. Euclidean distance matrices were generated for geographic distance between samples and Bray-Curtis dissimilarity matrices were calculated for plant composition within a 1 m radius of the sampling location. Using the "mantel" function from the vegan package [15], we found that the composition of bacteria and fungi immigrating into the soil surface was positively correlated with plant composition within 1 meter, where microbial communities became more similar in composition as the plant composition around the samples also increased in similarity (Bacteria: Mantel's r = 0.12, P value = 0.04; Fungi: r = 0.61, P value < 0.0001). Further, fungal, but not bacterial, composition was correlated with geographic distance between samples (Bacteria: Mantel's r =

0.06, P value = 0.13; Fungi: $r = 0.19$, P value < 0.001). Therefore, all downstream analyses were conducted using a partial Mantel test from the vegan package to remove variation in fungal composition explained by distance between samples. This previous analysis was then repeated except the radius at which plant composition was measured from the accumulation rate samples varied from 0.1 m to 4 m (0.1, 0.5, 0.75, 1, 1.25, 1.5, 2, 2.5, 3, 3.5, and 4 m). Note, however, that the measures of plant composition for the sampling locations closest to the ecosystem boundary were not independent along the same transect because circles of radii larger than 1 m overlapped. By graphing the strength of these correlations against the radii of the circles for which plant composition was measured, we identified the distance for which plant composition had the strongest influence on immigrating taxa.

Supplementary Information

Supplementary Figures

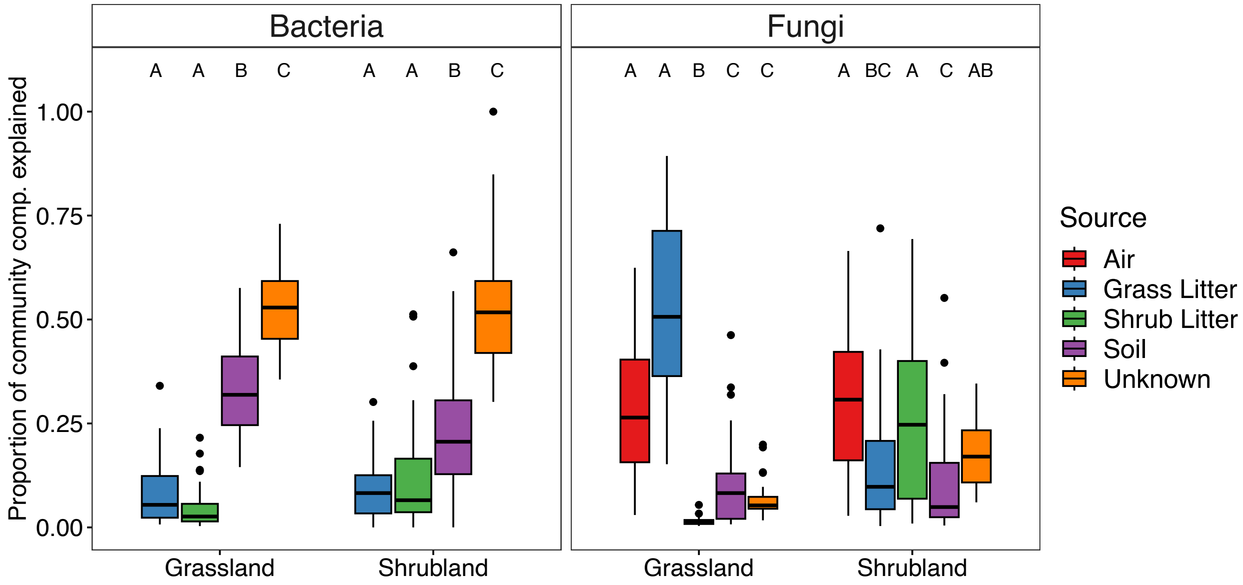

Figure S1. Proportion of bacterial and fungal community composition on the accumulation rate samples (open glass slides) attributed to distinct dispersal sources (air, grass or shrub litter, and surface soil). Letters indicate significantly pairwise differences within ecosystems using Dunn's multiple comparison post hoc test with Bonferroni correction. Bacteria were not successfully sequenced from the air samples. Therefore, the proportion of bacterial community composition explained by dispersal from air is included within the Unknown source.

Supplementary Information

Supplementary Tables

Table S1. ANCOVA results for the effects of ecosystem, time (days in the field), and time-by-ecosystem interaction on bacterial abundance on the death rate slides. Significant factors and p-values are bolded.

	Df	Sum Sq	*F* value	*P* value
**Ecosystem**	1	36.78	219.11	**< 0.001**
**Time**	1	11.12	66.23	**< 0.001**
Time x Ecosystem	1	0.33	1.98	0.17
Residuals	41	6.88		

Supplementary Information

Table S2. ANCOVA results for the effects of ecosystem, time (days in the field), and time-by-ecosystem interaction on bacterial accumulation or abundance on the accumulation rate slides. Significant factors and p-values are bolded.

	Df	Sum Sq	*F* value	*P* value
Ecosystem	1	1.31E+08	0.34	0.56
**Time**	1	2.71E+09	7.05	**0.01**
Time x Ecosystem	1	1.01E+08	0.26	0.61
Residuals	92	3.53E+10		

Supplementary Information

Table S3. PERMANOVA analysis for the effects of ecosystem, time (days in the field), and ecosystem-by-time interaction on community composition on the accumulation rate slides for A) bacteria and B) fungi. Significant factors and p-values are bolded. Asterisk indicates factor with a significant PERMDISP analysis (P ≤ 0.05).

	df	SS	MS	Pseudo-F	P(perm)	% Variance Explained
(A) Bacteria						
**Ecosystem**	1	0.79	0.79	2.16	**0.001**	2.9
**Time**	3	1.29	0.43	1.17	**0.007**	0.9
Time x Ecosystem	3	1.12	0.37	1.02	0.262	-
Residuals	65	23.78	0.37			96.2
Total	72	26.94				

	df	SS	MS	Pseudo-F	P(perm)	% Variance Explained
(B) Fungi						
**Ecosystem**	1	1.53	1.53	10.79	**0.001***	18.5
**Time**	3	1.23	0.41	2.89	**0.001**	7.1
**Time x Ecosystem**	3	0.65	0.22	1.53	**0.001**	4.0
Residuals	68	9.65	0.14			70.5
Total	75	13.05				

Supplementary Information

Table S4. Correlation between bacterial and fungal composition on the accumulation rate slides and plant community composition measured within a series of circles with increasingly large radii from the sample. Partial mantel tests were used to control for variation in microbial composition explained by geographic distance between samples. Correlation results reported as Mantel correlation coefficient (*r*) and significance (*P* value). Significant radii are bolded.

Organism	Plant sampling radius (m)	*r*	*P* value
Bacteria	0.1	0.12	0.052
	**0.5**	0.12	**0.047**
	**0.75**	0.12	**0.047**
	**1**	0.12	**0.040**
	1.25	0.11	0.055
	1.5	0.10	0.076
	2	0.08	0.100
	2.5	0.08	0.105
	3	0.08	0.107
	3.5	0.07	0.118
	4	0.07	0.114
Fungi	**0.1**	0.57	**<0.001**
	**0.5**	0.57	**<0.001**
	**0.75**	0.58	**<0.001**
	**1**	0.59	**<0.001**
	**1.25**	0.59	**<0.001**
	**1.5**	0.58	**<0.001**
	**2**	0.55	**<0.001**
	**2.5**	0.52	**<0.001**
	**3**	0.49	**<0.001**
	**3.5**	0.48	**<0.001**
	**4**	0.48	**<0.001**

Supplementary Information

References

1. Kimball S et al. Altered water and nitrogen input shifts succession in a southern California coastal sage community. *Ecological Applications* 2014;**24**:1390–1404. https://doi.org/10.1890/13-1313.1
2. Finks SS et al. Microbial community response to a decade of simulated global changes depends on the plant community. *Elementa* 2021;**9**:00124. https://doi.org/10.1525/elementa.2021.00124
3. Walters KE et al. Routes and rates of bacterial dispersal impact surface soil microbiome composition and functioning. *ISME Journal* 2022;**16**:2295–2304. https://doi.org/10.1038/s41396-022-01269-w
4. Khalili B et al. Optimization of a method to quantify soil bacterial abundance by flow cytometry. *mSphere* 2019;**4**:e00435-19. https://doi.org/10.1128/msphere.00435-19
5. Lane DJ et al. Rapid determination of 16S ribosomal RNA sequences for phylogenetic analyses. *Proceedings of the National Academy of Sciences* 1985;**82**:6955–6959. https://doi.org/10.1073/pnas.82.20.6955
6. Caporaso JG et al. Ultra-high-throughput microbial community analysis on the Illumina HiSeq and MiSeq platforms. *ISME Journal* 2012;**6**:1621–1624. https://doi.org/10.1038/ismej.2012.8
7. Looby CI, Maltz MR, Treseder KK. Belowground responses to elevation in a changing cloud forest. *Ecology and Evolution* 2016;**6**:1996–2009. https://doi.org/10.1002/ece3.2025
8. Bolyen E et al. Reproducible, interactive, scalable and extensible microbiome data science using QIIME 2. *Nature Biotechnology* 2019;**37**:852–857. https://doi.org/10.1038/s41587-019-0209-9

9. Callahan BJ et al. DADA2: High-resolution sample inference from Illumina amplicon data. *Nature Methods* 2016;**13**:581–583. https://doi.org/10.1038/nmeth.3869

10. Bokulich NA et al. Optimizing taxonomic classification of marker-gene amplicon sequences with QIIME 2's q2-feature-classifier plugin. *Microbiome* 2018;**6**:90. https://doi.org/10.1186/s40168-018-0470-z

11. Quast C et al. The SILVA ribosomal RNA gene database project: Improved data processing and web-based tools. *Nucleic Acids Research* 2013;**41**:590–596. https://doi.org/10.1093/nar/gks1219

12. Abarenkov K et al. The UNITE database for molecular identification and taxonomic communication of fungi and other eukaryotes: sequences, taxa and classifications reconsidered. *Nucleic Acids Research* 2024;**52**:D791–D797. https://doi.org/10.1093/nar/gkad1039

13. Barbour KM et al. Testing the contribution of dispersal to microbial succession following a wildfire. *mSystems* 2023;**8**. https://doi.org/10.1128/msystems.00579-23

14. R Core Team. A language and environment for statistical computing. 2020. Vienna, Austria: R Foundation for Statistical Computing, 2020.

15. Oksanen J et al. vegan: Community Ecology Package. 2022.

16. Clarke KR, Gorley RN. PRIMER v6: User Manual. *Plymouth Marine Laboratory, Plymouth* 2006.

17. Knights D et al. Bayesian community-wide culture-independent microbial source tracking. *Nature Methods* 2011;**8**:761–765. https://doi.org/10.1038/nmeth.1650

18. Mantel N. The detection of disease clustering and a generalized regression approach. *Cancer Research* 1967;**27**:209–220.

19. Sokal RR. Testing statistical significance of geographic variation patterns. *Systematic Zoology* 1979;**28**:227. https://doi.org/10.2307/2412528
